# Anterior retroperitoneal approach in constructing thymokidney organs in swine for xenotransplantation

**DOI:** 10.3389/frtra.2024.1473281

**Published:** 2024-11-14

**Authors:** M. Esad Gunes, Daniel H. Wolbrom, Sho Fujiwara, Susan Qudus, Alexander Cadelina, Greg Nowak

**Affiliations:** ^1^ Columbia Center for Transplantation Immunology, Columbia University, New York, NY, United States; ^2^ Department of Surgery, Columbia University, New York, NY, United States; ^3^ Division of Transplantation Surgery, Karolinska Institute, Stockholm, Sweden

**Keywords:** xenotransplantation, thymokidney, kidney, swine, thymus, tolerance, pig, transplantation

## Abstract

**Introduction:**

Thymokidneys (TK) have been constructed to transplant life-supporting kidney grafts containing donor thymic tissue to induce transplant tolerance. Historically, TKs were constructed by inserting pieces of thymus tissue under the kidney capsule using an intra-abdominal or posterior retroperitoneal (lateral/flank) approach. The intra-abdominal approach is technically easier but causes intra-abdominal adhesions and makes kidney procurement more challenging. The posterior retroperitoneal approach causes fewer complications, but thymus tissue implantation is technically demanding due to limited visibility and exposure of the kidney. We herein describe the anterior retroperitoneal approach that overcomes these challenges.

**Methods:**

8-week-old GalTKO-swine (*n* = 2) were sedated, intubated, and draped. Cervical thymus lobes were isolated and excised. Via a small midline abdominal incision, the peritoneum was dissected bilaterally from the abdominal muscles, identifying both kidneys without entering the peritoneal cavity. Multiple thymus pieces were inserted under the kidney capsule. After 8 weeks, TKs were recovered for flow cytometric and histopathological analysis.

**Results:**

In all kidneys, we successfully constructed TKs with functional thymus tissue under the kidney capsule, verified by histopathology and flow cytometry. No surgical complications were observed, and no adhesions were observed intra-abdominally nor around the kidney, as the peritoneum covered the implanted tissue.

**Conclusion:**

The anterior retroperitoneal approach to constructing thymokidneys is easy to perform, offers excellent kidney exposure, allows a larger volume of thymus tissue to be implanted, and decreases the risk of intra-abdominal adhesions. Such constructed TKs are easy to procure with minimal risk of injury to the vascularized thymus as the prerenal peritoneum covers it.

## 1 Introduction

Novel immunosuppression therapies and the generation of genetically modified swine have been able to circumvent hyperacute, acute, and subacute rejection in kidney xenotransplantation (KXTx), but chronic rejection remains a challenge (1, 2). One way in which chronic rejection may be overcome is through the development of xenotransplantation tolerance via thymus transplantation with appropriate immunosuppressive conditioning (3–5). In KXTx, vascularized thymic lobe (VTL) transplantation was utilized to achieve such an effect (5). However, VTL transplantation requires knowledge of and training in microsurgery and carries its own complications. To overcome these challenges, thymokidney (TK) organs have been constructed as an alternative approach, allowing the simultaneous transplantation of the life-supporting kidney graft along with vascularized donor thymic tissue.

Historically, TKs were constructed 6–8 weeks before transplantation to allow for neo-vascularization of the thymus tissue under the kidney capsule (6). A cervical thymectomy was performed, and pieces of autologous thymic tissue were prepared and inserted underneath the kidney capsule using either an intra-abdominal or posterior retroperitoneal (lateral/flank) approach (6–9). Of these, the intra-abdominal approach allowed for the insertion of multiple pieces of thymus and bigger total volumes of implanted tissue, but it led to intra-abdominal adhesions that presented a challenge during donor nephrectomy. The posterior retroperitoneal approach is an intra-abdominal adhesion-free procedure but does not allow for the insertion of multiple thymic fragments (6–9). In addition, the presence of perinephric adhesions still maintained a challenge in keeping the transplanted thymus intact during kidney procurement.

To overcome these issues, we have introduced an anterior retroperitoneal TK construction method to maximize the thymus volume in TKs, which is of crucial importance as thymus volume correlates with the rate of thymopoiesis in humans (10, 11), and to allow a less challenging donor nephrectomy after TK construction, with minimal to no intraabdominal adhesions. In this study, we evaluate the anterior retroperitoneal approach to create TKs in swine, which can be used in experimental models of operational transplant tolerance and potentially in future clinical xenotransplant trials.

## 2 Materials and methods

### 2.1 Animals

8-week-old female GalT-KO Sachs’ Miniature Swine (8–10 kg) (*n* = 2) were used for autologous TK (*n* = 4) construction (12, 13). The properties of these animals have been previously described (14, 15). Animals were housed in single cages following Columbia University Institute of Comparative Medicine (ICM) guidelines. All surgical and experimental procedures were approved by the Institutional Animal Care and Use Committee (IACUC) at Columbia University under the IACUC protocol number AC-AABN1550.

### 2.2 TK construction using the anterior retroperitoneal approach

To harvest the thymic tissue for autologous TK construction, we have opted to perform a total cervical thymectomy (subtotal thymectomy), as it was previously shown that a total thymectomy including also removal of intrathoracic thymus, is not necessary to promote engraftment of thymic tissue on the TK grafts (6).

**Figure 1:**
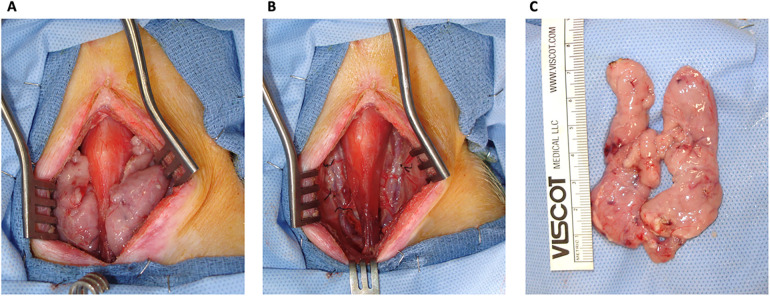
Cervical thymectomy. **(A)** A view of the thymic lobes before ligating the internal cervical artery and veins and recovery of the thymus. **(B)** No thymic tissue was left behind in the neck of the swine. **(C)** Cervical thymus *ex-situ*.

To do so, the animals were sedated, intubated, and draped. To recover the cervical thymus for TK construction, a midline incision on the neck was made between the sternum and mandibular angles. On both sides of the neck, the sternohyoid and sternocephalic muscles were retracted, and the cervical thymus lobes were visualized. On the medial sides, the cervical thymus lobes were dissected from the trachea, thyroid, and carotid sheaths. On the lateral sides, the thymus lobes were dissected from the muscles and external jugular veins. Dissection was continued posteriorly, and the thymus lobes were dissected from the prevertebral attachments. The internal cervical artery and vein of each thymus lobe were ligated using 2/0 silk. Both cervical thymic lobes were removed and placed in a cold saline solution (Figure 1). The neck incision was closed with 2/0 multifilament, absorbable suture, and staples.

**Figure 2:**
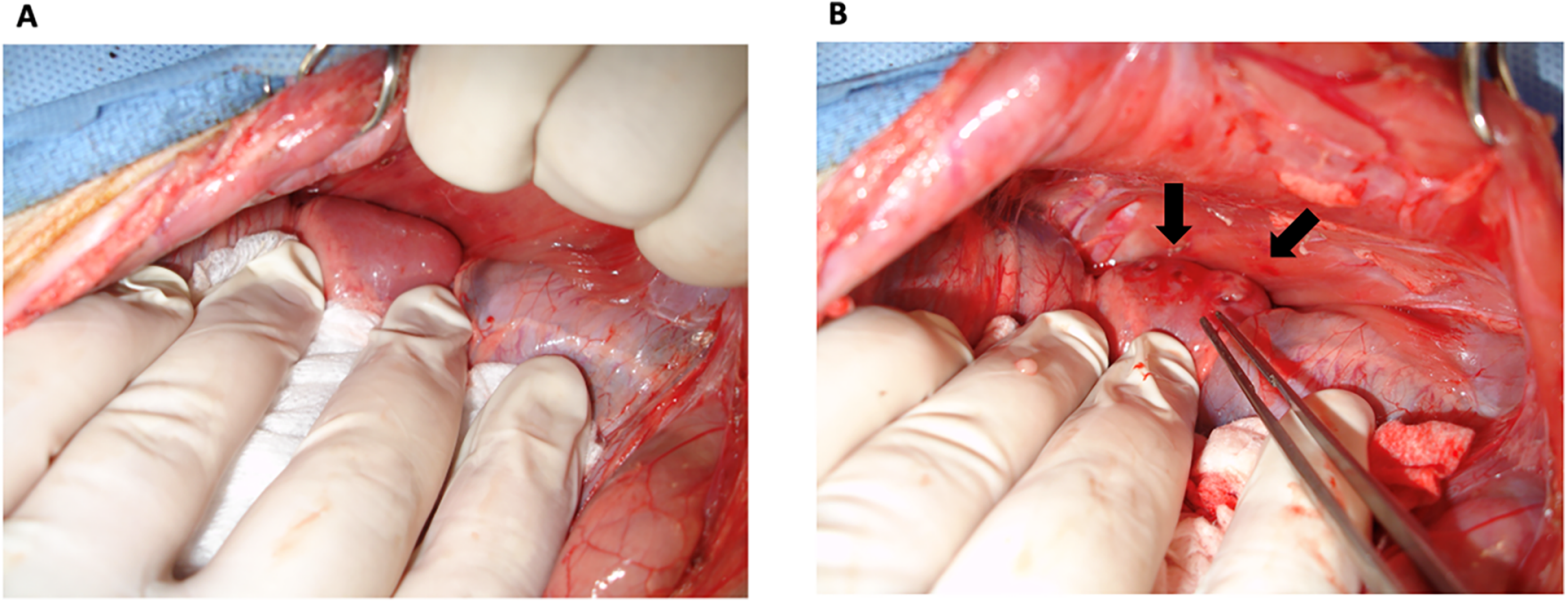
Thymokidney construction. TKs were constructed using anterior retroperitoneal approach. **(A)** A good view of the kidney was achieved without disrupting the peritoneum. **(B)** Thymic tissue was inserted underneath the kidney capsule. Arrows point to the inserted thymic tissue.

On the same animal, a small midline abdominal incision was made and dissected until the peritoneum was identified. The peritoneum was carefully dissected laterally through the fatty tissue between the transverse abdominal muscle and its fascia using a pair of cotton-tipped applicators and blunt dissection until the kidneys were identified. An incision was made on the lateral aspect of Gerota's fascia. The anterior surface of the kidneys was bluntly dissected from the peritoneum and Gerota's fascia, allowing direct visualization of the kidneys without disrupting the peritoneal cavity (Figure 2). Care was taken to minimize compression of the peritoneal cavity and the diaphragm so as not to compress the diaphragm during the surgery. On the back table, the cervical thymus was stripped of its capsule and cut into 1–2 mm^3^-sized pieces. Two to three 0.5 cm incisions were made on the kidney capsule with the tip of an 18G needle. A right-angle clamp was used to sweep the kidney capsule from the parenchyma in a circular motion to create space for thymic fragments, which were inserted underneath the kidney capsule using a right-angle clamp and fine forceps under magnification (Loupes 2.5×) (Figure 2). The peritoneum was carefully laid on the kidney, covering the insertion sites to prevent displacement of the thymic fragments. The abdominal fascia and skin were approximated with 2/0 PDS, 2/0 multifilament, absorbable sutures, and staples, respectively. One dose of cefazolin and SR buprenorphine were administered for perioperative antibiotic prophylaxis and postoperative pain control, respectively. The animals were followed for eight weeks before TK harvest (6).

### 2.3 Necropsy and tissue collection

**Figure 3:**
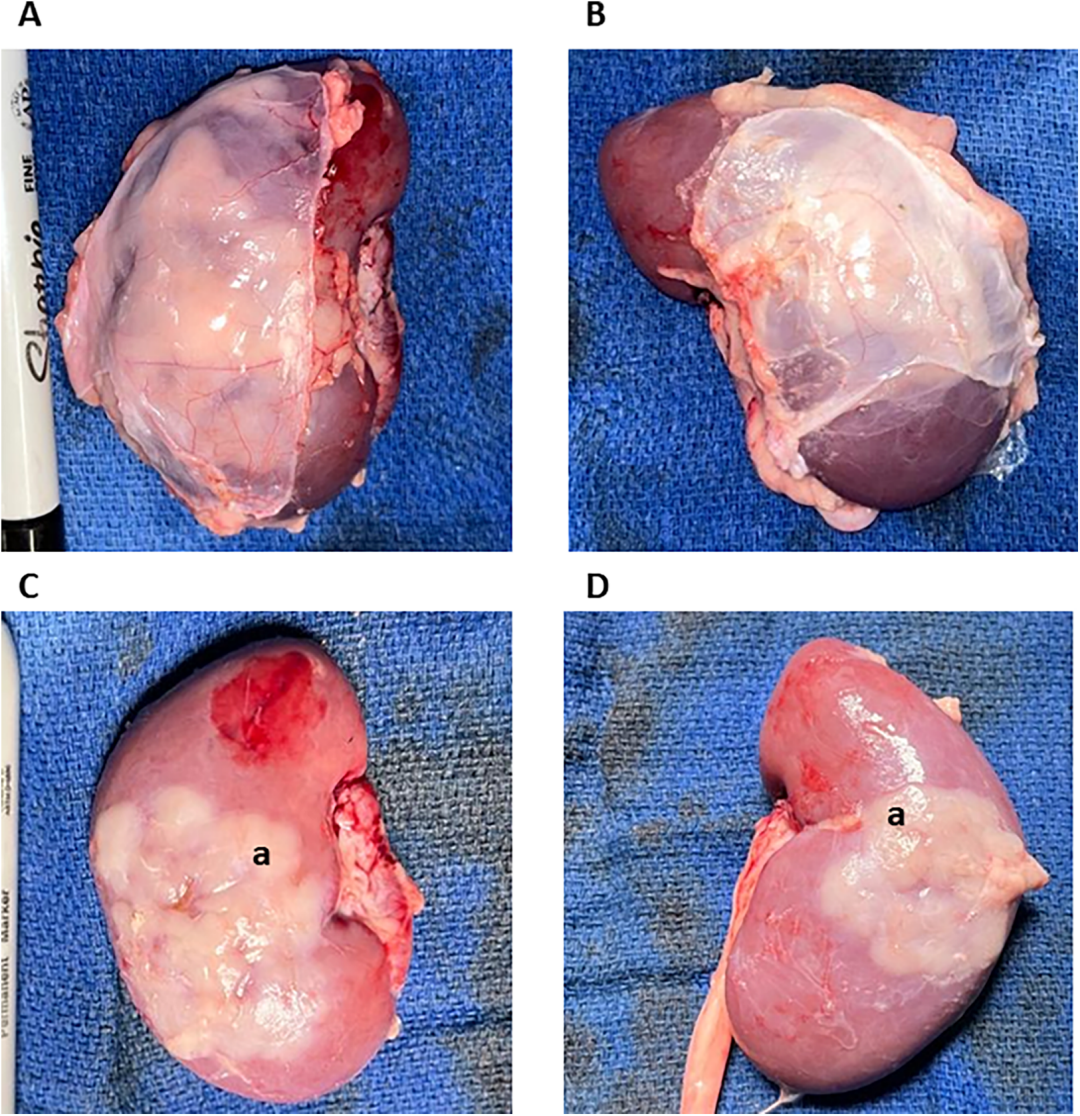
Macroscopic overview of the thymokidneys. TKs were harvested 8 weeks after construction. **(A,B)** represent the bilateral TKs harvested from animals 1 with their anterior peritoneum still attached. **(C,D)** Represent the bilateral TKs harvested from animals 1 after the removal of the peritoneum. Good thymic engraftment can be appreciated. (a.) Thymic Tissue.

The animals were euthanized eight weeks after TK construction using 100 mg/kg IV KCl. Usage of Euthasol (Virbag, Westlake, TX) was avoided as it negatively impacts tissue processing and histopathological and flow cytometric results. The thoracic and abdominal cavities were opened. The renal arteries and veins and the ureters were ligated. The TKs were recovered *en bloc* with the peritoneal layer covering the kidney. On the back table, the prerenal peritoneum was carefully dissected from the TK. Macroscopically, the thymus portion of the TKs under the kidney capsule was identified in all four constructed TKs (Figure 3). The TK samples were recovered as 1 cm^3^ pieces for histopathology. The thymus portion of the TKs was further stripped from the kidney for subsequent processing and flow cytometric analysis. A piece of the posterior surface of the kidneys and the native thoracic thymus were collected for histopathology and flow cytometric analysis to be used as negative and positive controls, respectively.

### 2.4 Tissue processing

Tissues were collected in RPMI-1640 (Cytiva, Marlborough, MA) and stored at 4°C before processing into a single-cell suspension for flow cytometry analysis. Tissue samples collected from all four TK, kidney, and native thoracic thymus were processed using the same technique described below.

On a tissue culture dish (Thermo Fischer, Waltham, MA), 10 mL of cold RPMI-1640 was added. A 70 µm strainer (Alkali Scientific, Fort Lauderdale, FL) was placed in the dish. A piece from a tissue sample was cut into smaller pieces and placed inside the 70 µm strainer. The end of a 20 mL syringe (McKesson, Irving, TX) was used to crush the tissue through the strainer. The cells were collected from the dish into a 50 mL conical and washed with HBSS (Cytiva, Marlborough, MA, USA). Red blood cells were lysed using ACK lysing buffer (Thermo Fischer, Waltham, MA, USA). The cells were washed, resuspended in HBSS, and stored at 4°C for further processing and analyses.

### 2.5 Flow cytometry

Our method of sinxgle-cell analysis via flow cytometry has previously been detailed (16). Shortly, the single cell suspensions from the tissues were Fc-blocked with 5 µl of heat-inactivated normal porcine serum (Thermo Fisher, Waltham, MA) and stained with mouse-anti-pig CD3 (Clone: BB23-8E6-8C8), CD4 (Clone: 74-12-4), and CD8α (Clone: 76-2-11) (BD, Franklin Lakes, NJ) antibodies for 30 min at 4°C. The tubes were washed twice with FACS Buffer (BD, Franklin Lakes, NJ). DAPI (Abcam, Waltham, MA) was added before acquisition to distinguish dead cells. The data was acquired on a 5 Laser Cytek Aurora Flow Cytometer (Cytek, Fremont, CA) and analyzed using FCS Express (*de novo* Software, Pasadena, CA). Live and singlet cells were selected and plotted using CD4 and CD8 to select CD4+CD8+ DP and CD4-CD8- DN thymocytes. CD4+CD8+ DP and CD4-CD8- DN thymocytes were further gated using CD3 to indicate the presence of CD3-CD4+CD8+ DP and CD3+CD4-CD8- thymocytes, which are specific cell phenotypes found only in the thymus.

### 2.6 Histopathology

Tissues were collected and fixed in 10% buffered formalin (McKesson, Irving, TX) for 24–48 h for histopathology studies. Samples were processed and stained with H&E by the Molecular Pathology Core at Columbia University. Slides were analyzed using Aperio ImageScope (Leica Camera, Teaneck, NJ).

## 3 Results

### 3.1 Construction of thymokidneys using the anterior retroperitoneal approach

**Figure 4:**
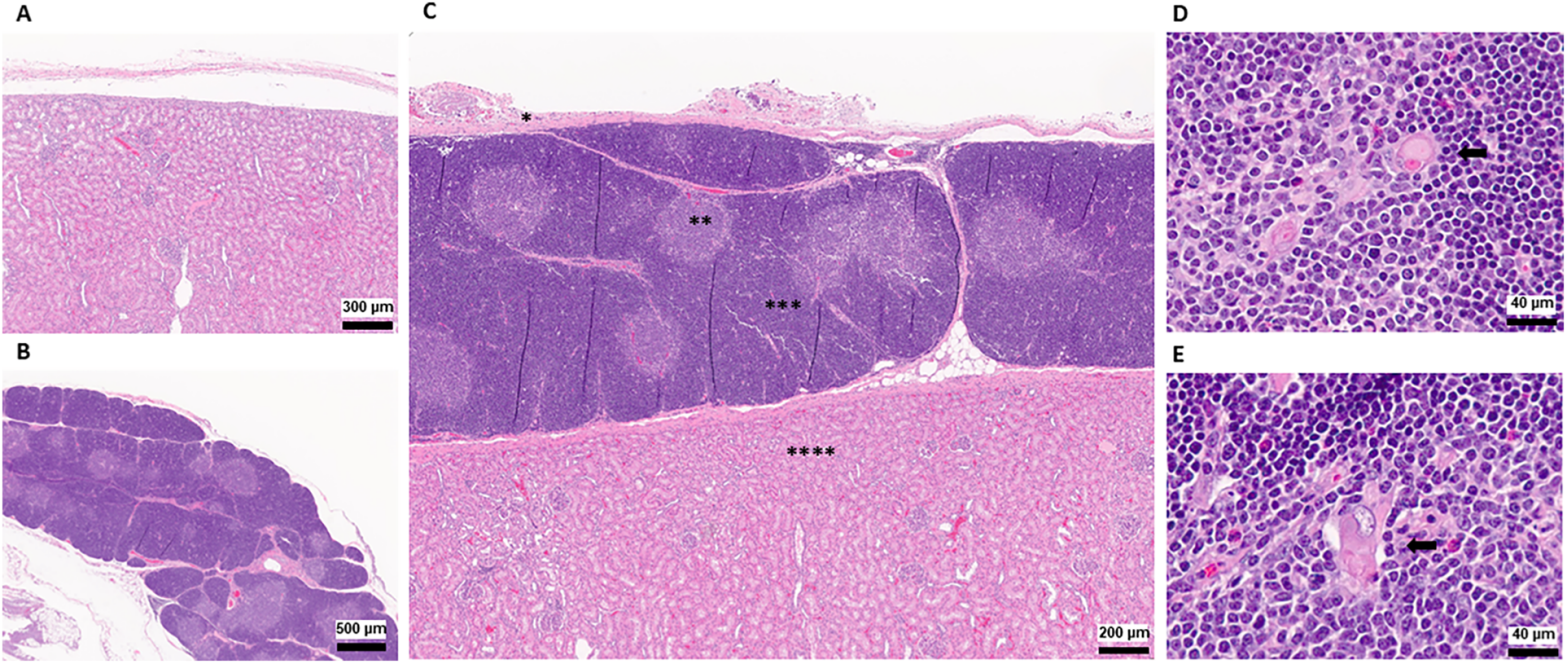
Histopathology of thymokidneys. **(A)** A histopathological view of the posterior aspect of the TK, recovered as a negative control. **(B)** A histopathological view of the naïve thoracic thymus, recovered as a positive control. **(C)** A histopathological view of the TK recovered 8 weeks after TK construction. * Kidney Capsule, **Thymic Medulla, ***Thymic Cortex, ****Kidney Parenchyma **(D)** A 64× magnified view of the naïve thoracic thymus medulla recovered at the time of the necropsy. **(E)** A 64× magnified view of the TK thymus medulla recovered at the time of the necropsy. Black arrows indicate Hassall's Corpuscles.

**Figure 5:**
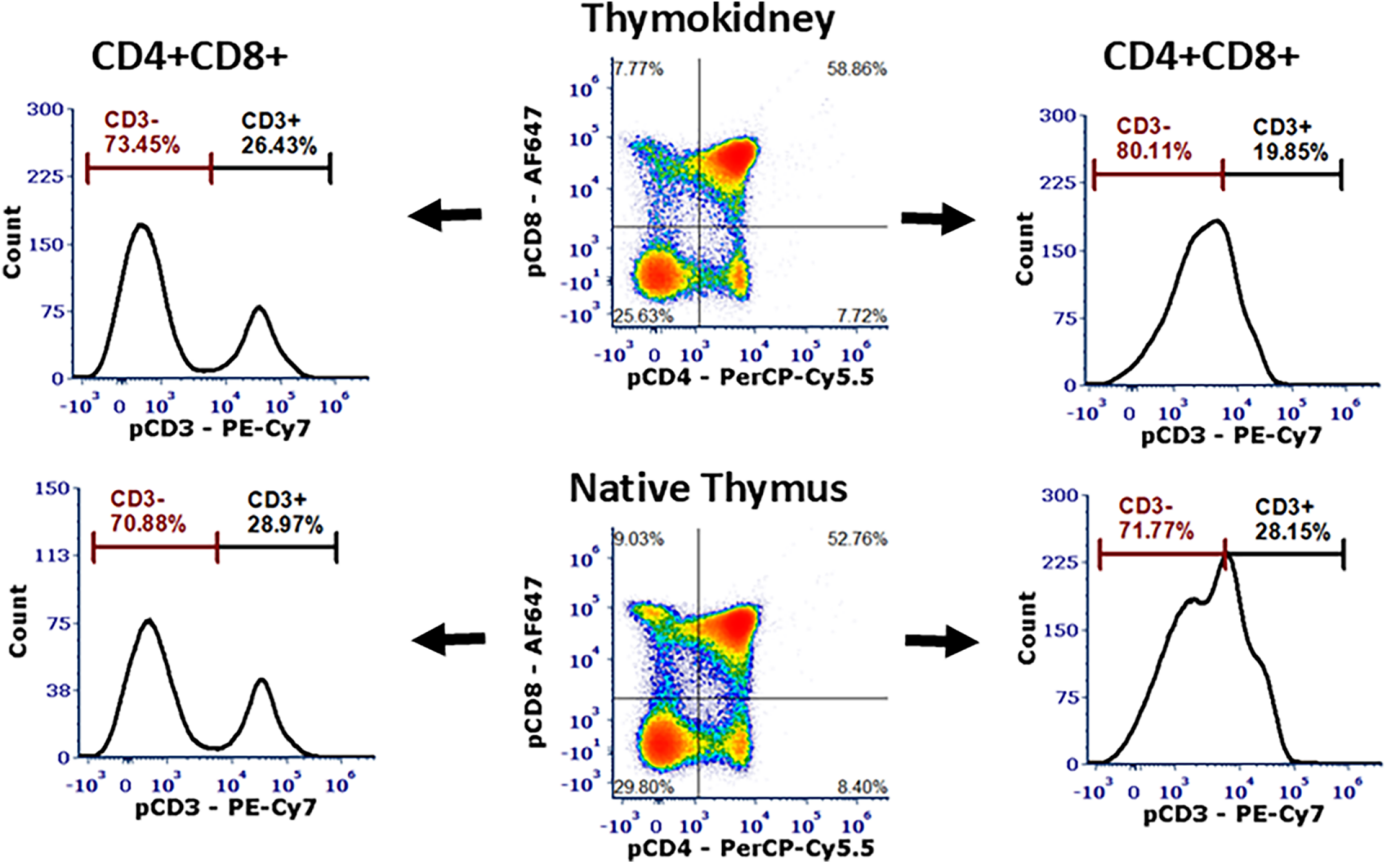
Flow cytometric analysis of the thymokidneys. Single-cell analysis of the thoracic thymus and a thymokidney from the same animal verified ongoing thymopoiesis in both tissues with the presence of CD3-CD4+CD8+ DP cells and CD3+CD4-CD8- DN cells.

We have constructed bilateral TKs (*n* = 4) in 2-month-old GalT-KO miniature swine (*n* = 2) using the anterior retroperitoneal approach. Both animals underwent total cervical thymectomy and subsequent autologous TK construction without any intra or postoperative complications (Figures 1, 2). Postoperative care and recovery were no different than the intraabdominal or posterior retroperitoneal approach. We allowed the thymus to neovascularize and engraft for a period of eight weeks based on the literature (6). Thereafter, the animals were euthanized, and tissues were collected for histopathologic and flow cytometric analysis. Our approach yielded successful engraftment of the thymus tissue, which covered approximately 30%–50% of the anterior surface of the kidneys (Figure 3), as confirmed macroscopically and by histopathology and flow cytometric analysis (Figures 4, 5).

### 3.2 The anterior retroperitoneal approach allowed better visualization of the kidneys and easier thymic tissue implantation

During the TK construction, our anterior retroperitoneal approach allowed for greater visualization of the entire kidney compared to the posterior retroperitoneal approach (Figure 2). This level of kidney visualization was previously available only through the intra-abdominal approach and gave us access to the entire anterior surface of the kidney for subsequent thymic tissue implantation. During thymic tissue insertion under the kidney capsule, we were able to insert thymus pieces that covered around 30%–50% of the anterior surface of one kidney, which amounted to approximately 3–4 cm^3^ (1/2–2/3 of one cervical thymus lobe) (Figures 1, 2). Based on our experience, this represented an increase of approximately 4-fold more thymic tissue implantation compared to the posterior retroperitoneal approach.

### 3.3 Retrieval of the thymokidneys without adhesions

We procured the TKs *en bloc* with the anterior peritoneum to avoid disruption of the thymic tissue during procurement (Figure 3). We encountered no adhesions either intra-abdominally or around the kidney, as the peritoneum gently covered the implanted thymus. Even though we performed a double-side peritoneum dissection from the abdominal wall, there were no signs of any complications related to it. At the back table, we carried out a gentle dissection of the prerenal peritoneum. After removing the prerenal peritoneum, macroscopically, all 4 TKs had very successful engraftment of the thymic tissue, covering 40%–60% of the anterior surface of the kidneys (Figure 3). We collected tissues for flow cytometry and histopathology, as described below.

### 3.4 Histopathological and flow cytometry analysis

At the time of necropsy, we collected biopsies from the thoracic thymus and posterior surface of the kidneys to be used as positive and negative controls, respectively. The corticomedullary thymic architecture in the TKs was similar to the native thoracic thymic tissue (Figure 4). Furthermore, Hassall's corpuscles were noted in the medulla of the TK, indicating the presence of a healthy and functioning thymus tissue (Figure 4D) (17, 18). In single-cell phenotype analysis using flow cytometry, the presence of thymus tissue and thymopoiesis was detected by identification of CD3-CD4+CD8+ DP and CD3+CD4-CD8- DN cells. All four thymus tissues recovered from TKs, as well as the native thoracic thymus, demonstrated 70%–80% CD3-CD4+CD8+ DP cells and 15%–30% CD3+CD4-CD8- DN cells (Figure 5).

## 4 Discussion

Xenotransplantation is a rapidly developing research area, with pre-clinical studies in deceased human donors already underway (19, 20). Recently, two human heart transplants were reported as an important step toward clinical trials (21). The development of operational xenotransplantation tolerance would undoubtedly boost the xenotransplantation efforts in clinical trials. To achieve tolerance, simultaneous vascularized donor thymus transplantation, together with the target organ, was proposed (3). An example of such an approach is the use of TK, which is composed of the donor's thymus and life-supporting kidney in one vascularized graft, prepared at least six weeks before transplantation (6). Even though TK construction has been performed for over 20 years, we believe it is important to optimize and standardize TK construction to maximize its benefits across xenotransplantation barriers and allow other researchers to reproduce and research the TK xenotransplantation model readily and robustly.

Historically, the first reported TK in swine was constructed using the intra-abdominal approach, which allowed for greater visualization of the kidneys and large-volume thymic tissue implantation (6). However, this technique was abandoned due to intra-abdominal adhesions that were very challenging during donor nephrectomy, and therefore the posterior retroperitoneal approach was adopted (9). However, the posterior retroperitoneal approach offers limited visibility and exposure of the kidney, leading to the implantation of much smaller volumes of thymus tissue under the kidney capsule. Retrorenal adhesions also proved to be challenging, with a high risk of thymus tissue injury during kidney procurement. On the other hand, it has been shown that the volume of transplanted thymus corresponds to the rate of posttransplant thymopoiesis in TK (22). To overcome these challenges and standardize TK construction, we have proposed and developed the anterior retroperitoneal approach presented here, which combines the superiorities of each previous technique, where an excellent kidney visualization and a large volume of thymic tissue implantation with minimal risk of intra-abdominal adhesions is readily achievable, and the kidney procurement is less challenging with minimal risk of thymic injury. Dissection of the peritoneum from the fascia of the transverse abdominal muscle is easy as pigs have a high body fat content, which allows a safe blunt dissection.

The anterior retroperitoneal approach allowed for the implantation of a larger volume of thymic tissue, covering 30%–50% of the anterior surface of the kidney in our cohort, and this percentage can be easily increased and even doubled by using the posterior surface of the kidney which is easy to access via the anterior retroperitoneal approach. By using this approach, we increased the volume of implanted thymic tissue almost 2–3 fold compared to the posterior retroperitoneal approach.

After anterior retroperitoneal implantation of the tissue under the kidney capsule, the peritoneum was gently laid on the kidney to firmly cover the insertion areas by the positive pressure created by the peritoneal cavity. This mechanism prevented the displacement of inserted thymic tissue, which was previously done using a suture placed on the insertion sites. Therefore, suturing of insertion holes in the capsule is not needed in the anterior retroperitoneal approach, and the use of the anterior retroperitoneal technique helps to ensure good results despite possible implantation imperfection while establishing the TK construction technique from the beginning.

Most importantly, in addition to allowing large thymus volume implantation, the anterior retroperitoneal technique also decreases the risk of perinephric or intra-abdominal adhesion formation. During the TK retrieval in our cohort, no intra-abdominal nor perinephric adhesions were observed as the thymus was gently covered by the peritoneum, procured *en bloc* with the TK. Thus, the anterior retroperitoneal approach leaves the abdominal cavity intact, making any subsequent organ procurement with or without *in situ* cold organ perfusion or another intra-abdominal surgery after the construction of TK, such as thymic implantation of the islet to construct islet-thymo-kidney, relatively easy to perform.

Procurement of the TK *en bloc* with the peritoneum also prevents any potential injury to the thymic tissue. Such damage was especially encountered in the intra-abdominal approach, where meticulous dissection was being performed due to adhesions, as well as in the posterior retroperitoneal approach, where the thymus would adhere to the perinephric fat, requiring further dissection with significant risk for implanted thymus tissue damage. The operation time required for bilateral TK construction using the anterior retroperitoneal approach was similar to the posterior retroperitoneal approach, in which the animal had to be repositioned and re-draped, and to the intra-abdominal approach, in which the abdominal cavity had to be opened, and the kidneys needed to be dissected. The recovery and postoperative care after the anterior retroperitoneal approach were the same as after the intra-abdominal or posterior retroperitoneal approach, with no differences in the peri- and postoperative care.

In our pig-to-baboon xenotransplantation studies, based on anatomical advantages, the left donor kidney is transplanted to the recipient's right flank to minimize surgical complexity and maximize success. Compared to the posterior retroperitoneal approach, the anterior retroperitoneal approach allows us to create the thymus on the anterior surface of the left kidney, which, when transplanted in the right flank, has the thymus portion facing posteriorly. We believe that the positioning of the thymus posteriorly will prevent or minimize its interaction with the recipient's intrabdominal organs, causing fewer adhesions. On the other hand, if the right kidney is utilized, the porcine peritoneum harvested en-block can prevent the interaction of the thymus, facing anteriorly, and the intraabdominal organs.

In conclusion, we have successfully developed an anterior retroperitoneal approach to construct TKs in swine, which can be used in future experimental and clinical xenotransplant trials. Our approach is easy to perform and offers excellent kidney exposure, allows a large volume of thymus tissue implantation, and decreases the risk of intra-abdominal adhesions, making TK retrieval during donation easier and safer than after the intra-abdominal TK construction. Additionally, we observed no complications from bilateral peritoneum dissection were observed, indicating that the technique is safe for the TK donor and would also allow the donor to recover with fewer complications as the transplant time may vary from weeks to months.

## Data Availability

The original contributions presented in the study are included in the article/Supplementary Material, further inquiries can be directed to the corresponding author.
